# Assessing Disparities in Depression and Loneliness Between Older Immigrants and Natives: A SHARE Data Analysis for Luxembourg & Switzerland

**DOI:** 10.1002/alz70860_103879

**Published:** 2025-12-23

**Authors:** Ana Carolina Teixeira Santos, Joana Carvalheiro, Anja K. Leist

**Affiliations:** ^1^ University of Luxembourg, Esch‐sur‐Alzette, Luxembourg; ^2^ University of Glasgow, Glasgow, Glasgow, United Kingdom

## Abstract

**Background:**

Older immigrants face unique challenges impacting mental health, including modifiable risk factors for dementia, such as depression and loneliness. These issues are closely tied to social determinants of health, like income, education, social engagement, and housing conditions. The interaction between these determinants and migrant status may further increase vulnerability. Luxembourg and Switzerland, with significant aging immigrant populations, provide ideal contexts for exploring these phenomena.

This study examines disparities in depression and loneliness between older immigrants and non‐immigrants in Luxembourg and Switzerland, while exploring the influence of social determinants. We hypothesize that immigrants report poorer mental health outcomes, including higher levels of depression and loneliness.

**Methods:**

This study uses data from the Survey of Health, Ageing and Retirement in Europe (SHARE) Wave 9, including immigrant and non‐immigrants in Luxembourg (N_immigrants = 264, N_non‐immigrants = 546; mean age = 72.30 ±7.99, females = 54.81%) and Switzerland (N_immigrants =289, N_non‐immigrants = 1539; mean age = 74.20 ±9.41, females = 55.15%). Mental health was assessed using the EURO‐D depression scale and the Three‐Item Loneliness Scale to examine differences in migrant status, as well as the influence of individual and social determinants of health (i.e., age, income, education, marital status, social network, housing conditions, and the length of time since migration) in interaction with migration status, through linear regression models adjusted for age.

**Results:**

Immigrants reported poorer mental health compared to non‐immigrants, with higher levels of depression and loneliness across both countries (*p* < 0.02). Furthermore, the influence of sex and area of residence (ranging from rural regions to major cities) on depression varied depending on migration status, while social network satisfaction showed distinct associations with loneliness across these groups (*p* < 0.05).

**Conclusions:**

This study highlights significant disparities between older immigrants and non‐immigrants, with depression and loneliness emerging as key components of mental health in this population. The findings underscore the importance of considering factors such as sex, area of residence, and social network when developing policies and interventions to support the mental health of older immigrants. Addressing these mental health challenges may help mitigate the risk of dementia in this vulnerable group.

